# Triple-Negative Breast Cancer: A Review of Conventional and Advanced Therapeutic Strategies

**DOI:** 10.3390/ijerph17062078

**Published:** 2020-03-20

**Authors:** Mauricio A. Medina, Goldie Oza, Ashutosh Sharma, L.G. Arriaga, José Manuel Hernández Hernández, Vincent M. Rotello, Jose Tapia Ramirez

**Affiliations:** 1 Department of Nanoscience and Nanotechnology, CINVESTAV, Zacatenco, Avenida Instituto Politécnico Nacional 2508, Mexico City 07360, Mexico; 2 Centro de Investigación y Desarrollo Tecnológico en Electroquímica (CIDETEQ), Parque Tecnológico Querétaro s/n, Sanfandila. Pedro Escobedo, Querétaro 76703, Mexico; larriaga@cideteq.mx; 3 Tecnologico de Monterrey, School of Engineering and Sciences, Campus Queretaro, Av. Epigmenio González No. 500, Fracc. San Pablo, Queretaro 76130, Mexico; asharma@tec.mx; 4 Department of Cell Biology, CINVESTAV, Zacatenco, Avenida Instituto Politécnico Nacional 2508, Mexico City 07360, Mexico; 5 Department of Chemistry, University of Massachusetts, 710 North Pleasant Street, Amherst, MA 01003, USA; rotello@umass.edu; 6 Department of Genetics and Molecular Biology, CINVESTAV, Zacatenco, Avenida Instituto Politécnico Nacional 2508, Mexico City 07360, Mexico

**Keywords:** nanomedicine, triple negative breast cancer, artificial intelligence, theranostics, immunotherapy

## Abstract

Triple-negative breast cancer (TNBC) cells are deficient in estrogen, progesterone and ERBB2 receptor expression, presenting a particularly challenging therapeutic target due to their highly invasive nature and relatively low response to therapeutics. There is an absence of specific treatment strategies for this tumor subgroup, and hence TNBC is managed with conventional therapeutics, often leading to systemic relapse. In terms of histology and transcription profile these cancers have similarities to BRCA-1-linked breast cancers, and it is hypothesized that BRCA1 pathway is non-functional in this type of breast cancer. In this review article, we discuss the different receptors expressed by TNBC as well as the diversity of different signaling pathways targeted by TNBC therapeutics, for example, Notch, Hedgehog, Wnt/b-Catenin as well as TGF-beta signaling pathways. Additionally, many epidermal growth factor receptor (EGFR), poly (ADP-ribose) polymerase (PARP) and mammalian target of rapamycin (mTOR) inhibitors effectively inhibit the TNBCs, but they face challenges of either resistance to drugs or relapse. The resistance of TNBC to conventional therapeutic agents has helped in the advancement of advanced TNBC therapeutic approaches including hyperthermia, photodynamic therapy, as well as nanomedicine-based targeted therapeutics of drugs, miRNA, siRNA, and aptamers, which will also be discussed. Artificial intelligence is another tool that is presented to enhance the diagnosis of TNBC.

## 1. Introduction

Breast cancer is a pathology that emerges from the breast tissue, especially milk duct (ductal carcinoma representing 80% of the cases) as well as the lobules. The cancer emerging from the ductile region is known as ductal carcinoma while those emerging from the mammary lobules are known as lobular carcinomas [1].

As per the World Health Organization (WHO), breast cancer (BC) leads to mortality of women worldwide (age group: 20–59 years) (World Health statistics 2013). According to the global cancer project (GLOBOCAN 2012) breast cancer is considered as second most commonly occurred pathology in the world [2]. Breast cancer is a commonly occurring disease in less-developed and industrialized countries as well as the second notable cause of mortality in Europe and the United States after lung cancer [2,3].

In everyday medical practice, breast cancer diagnosis relies on three different types of analysis: (A) clinical examination; (B) radiological/image examinations (that includes mammography, magnetic resonance imaging (MRI) ultrasonography etc.) and (C) immunohistopathological examinations [4]. Employing all these tools, the clinical oncologist can stage the disease using TNM classification [5] and reviewing the guidelines established in rigorous clinical trials, although, they can also use genetic profiling tests such as MammaPrint [6] and Oncotype DX [7] to understand disease prognosis better.

However, two decades ago, before the arrival of the current therapeutics, something was still missing and a stronger diagnostic tool was needed. New advances in personalized medicine emerged with the technology of microarrays and the discovery of molecular profiles.

There are different histological subtypes of cancer (including breast cancer) within the same organ or tissue, but biopsies are heterogeneous because they include diverse types of cells. Classification of the different kinds of invasive carcinoma of breast cancer has been pursued, yet the clinical importance of its classification is limited because different breast cancer patients have a wide variety of different molecular profiles.

Breast cancer includes molecular biomarkers [8] include:

(1) ERα+ (estrogen receptor a-positive);
(2) PR+ (progesterone receptor-positive);
(3) HER-2 (human epidermal growth factor receptor-2);
(4) EGFR (epidermal growth factor receptor) 45%–70% of Triple-negative breast cancer (TNBC) patients show this biomarker [9];
(5) CK5/6;
(6) VEGF (vascular endothelial growth factor);
(7) KI67.

Currently, due to microarray technology, there is a better understanding of the molecular heterogeneity in tumors [10], aiding in the quantification of thousands of gene expression changes.

The classification of breast cancer cell types is considered as below:

(1) Luminal A subtype, ERα+/PR+ or −/HER-2-;
(2) Luminal B subtype, ER+/PR+/HER-2+;
(3) HER-2 enriched subtype ER- and or/PR−/HER-2+;
(4) Basal-like subtype ER^−^ and/or PR^−^, HER2^−^, CK5/6+, CK14+, CK17+ and EGFR+;
(5) Normal breast-like type (ER− and/or PR^−^, HER2^−^, CK5/6^−^, CK14^−^, CK17^−^, EGFR^−^) [11,12,13,14].

Moreover, a subpopulation is described due to the Ki-67 index too [15]. Therefore, molecular classifications are essential to provide personalized medicine and thus helps in selecting more specific drug according to the molecular signature markers of the tumor. Jézequel et al. found 4 TNBC subtypes:

(1) Luminal androgen receptor-AR (LAR);
(2) Mesenchymal (MES);
(3) Basal-like immune-suppressed (BLIS);
(4) Basal-like immune-activated (BLIA).

Their results suggest that BLIA tumor prognosis is improved as compared to BLIS tumors [16], [17]. Later, Lehman researchers described six subtypes of TNBC, which include [18]:

(1) Basal-like namely, BL1 and BL2;
(2) MES;
(3) MES stem-like;
(4) immunomodulatory (IM);
(5) LAR subtype.

The different subtypes of TNBC correlate well with the different chemotherapeutic responses as per retrospective studies [19]. Unfortunately, these molecular classifications have not been shown to improve survival in hospital practice and current treatments.

## 2. Signaling Pathways Involved in Triple-Negative Breast Cancer (TNBC) Therapeutics

### 2.1. Notch Signaling Pathway

Thomas Hunt Morgan described in 1917 a family of transmembrane ligands and receptors called Notch [20]. This signaling pathway has a pertinent role in cell proliferation as well as differentiation and, the elevated expression of a group of signaling molecules belonging to this pathway is correlated with the poorest outcome of patients [21]. The pathway comprises of 4 Notch receptors namely, Notch-1, 2, 3 and 4) as well as 5 ligands namely, Jagged-1, Jagged-2, Delta-like 1, Delta-like 3 and Delta-like 4. There are reports that confirms overexpression of Delta 1 and Jagged 1 in breast cancer [22,23,24], while Notch-1 also plays an important role in the origination of human mammary tumor in the form of a downstream effector of oncogenic Ras [25]. To date, Notch 1 has been relevant to the participation of the Notch channel in different types of hematological malignancies [26], pancreatic cancer [27] and many others. Several studies suggest that Notch-3 and Notch-4 are related to survival and proliferation of tumors. In contrast, overexpression of Notch-2 in the context of the TNBC MDA-MB-231 cell line appears to act as a protective factor [28]. 

**Figure 1:**
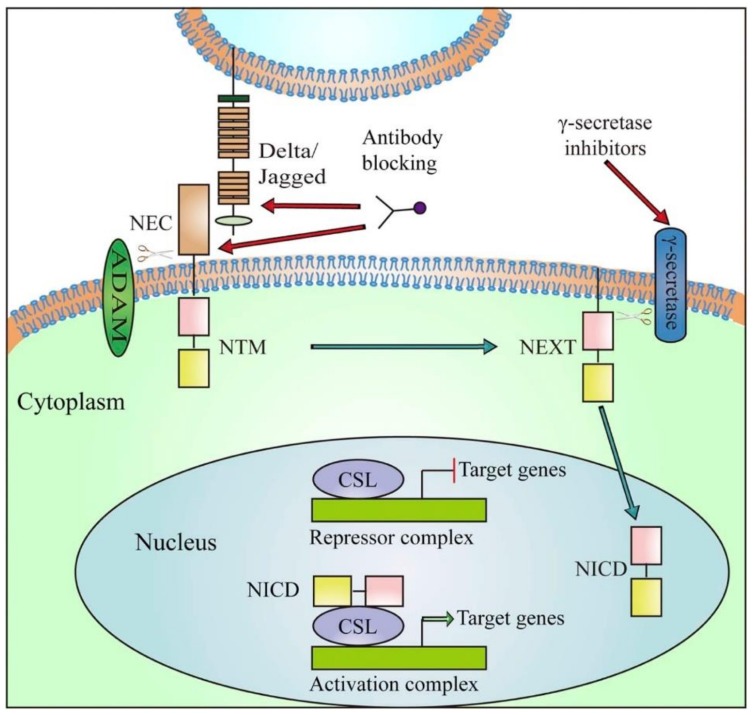
Diagram of Notch receptor activation and therapeutic target in clinical development. Notch signaling is initiated by ligand binding to Notch receptor, which undergoes a two-step proteolytic cleavage by ADAM family proteases and γ-secretase, releasing the Notch intracellular domain (NICD). The NICD translocates to the nucleus where it binds to CSL and converts the complex from a repressor to an activator of Notch target genes. Notch signaling could be inhibited by two major classes of Notch inhibitors: γ-secretase inhibitors and monoclonal antibodies directing against Notch receptors or ligands. Abbreviations: NEC, Notch extracellular subunit; NTM, Notch transmembrane fragment; NEXT, Notch extracellular truncated; CSL, C protein binding factor 1/Suppressor of Hairless/Lag-1; NICD, Notch Intracellular Domain. Reproduced with permission from Yuan X, Wu H, Xu H, Xiong H, Chu Q, Yu S. Notch signaling: An emerging therapeutic target for cancer treatment. Cancer Letters. 2015, 369, 20–27 [34].

Since Notch receptor and ligand overexpression is linked with TNBC, researchers believe that the receptor can be targeted by a monoclonal antibody (mAb) [29]. The current studies about inhibition of Notch-1 signaling by mAbs have shown effectiveness in reducing the expression of HES and HEY-L families in the MDA-MB-231 * TNBC cell lines (thus showing decrease in cell proliferation and increase in the induction of apoptosis [30]. Additionally, DLL4 (Delta-like ligand 4 Notch ligand) mAb therapy is effective for the treatment of TNBC [31]. Notch signaling in many transcription factors codifies genes related to tumorigenesis, for example the HES family, HEY family, Akt, p53, VEGF and PI3K-AKT-mTOR among others [32,33] (See Figure 1). Medications that interrupt Notch signaling pathway act at the level of the second proteolytic cleavage in the cell cytoplasm by blocking the multimeric γ-secretase complex and hence these drugs are known as γ-secretase inhibitors (GSIs) [33]. Unfortunately, most of the drugs that act by blocking the Notch pathway have not met expectations required for approval by the FDA (Food and Drug Administration).

### 2.2. Hedgehog Signaling Pathway

Sonic Hedgehog (Shh) [35] network morphogenes have an impact on cancer stem cell (CSC) maintenance, polydactyly syndromes, and basal cell carcinoma (Gorlin syndrome), with recent studies suggest that they are altered in clinical samples of several human cancers including breast cancer cell lines [36,37]. Hedgehog signaling involves three ligands:

(1) Sonic (SHH) highly expressed during embryogenesis;
(2) Indian (IHH) [38] mostly expressed in hematopoietic cells, endochondral skeleton, and cartilage;
(3) Desert (DHH) [39] exhibit expression in the peripheral nervous system and testes, in fact, mutations of the DHH gene could lead to pure gonadal dysgenesis (PGD) [40].

The Hedgehog signaling pathway is involved in the invasion of cancer cells, metastasis, and resistance of drugs as well as tumor recurrence cancer after therapy [41]. Kaplan–Meier survival studies indicate that overexpression of Shh is responsible for poor prediction of mortality in the breast cancer patients and especially, TNBC patients. SHH has an important role in the erroneous origin of malignancy in breast cancer because it maintains abnormal proliferation and promotes invasion to other tissues (metastasis). Researchers have designed novel experimental drugs namely, Thiostrepton, whose pharmacological action consists of targeting the sonic Hedgehog signaling, Thiostrepton suppresses the population of CD44+/CD24− cancer stem cells (CSCs) of TNBC cell lines [42]. Nevertheless, it is necessary to clarify the role of the Hedgehog pathway in breast CSCs [43] which has not been determined yet [44,45]. As a result, there are few drugs authorized by the FDA to date to address this pathway such as Vismodegib, which is used in basal cell carcinomas [46]. However, more research is needed for SHH signaling potentially leading to the design of new prevention tools and novel molecular markers for evaluation of recurrence, survival, and prognosis.

### 2.3. Wnt/β-Catenin Pathway

**Figure 2:**
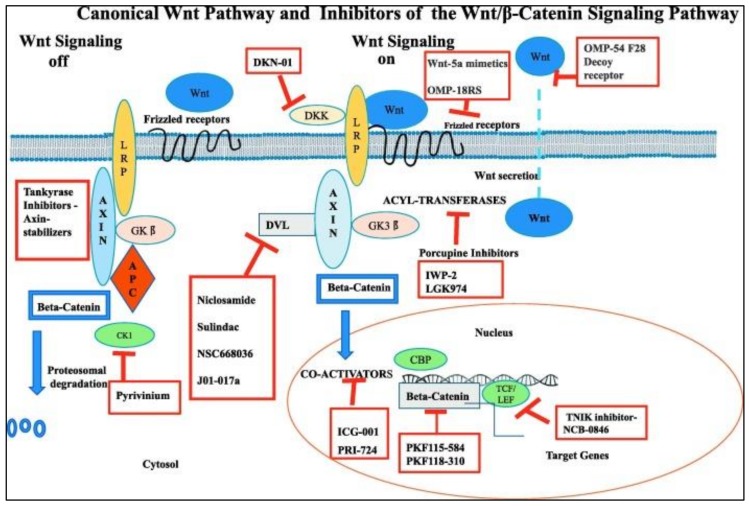
Canonical Wnt Pathway and Inhibitors of the Wnt/beta-Catenin Signaling Pathway schematic representation of the Canonical Wnt Pathway and pharmacologic inhibitors of the Wnt/beta-catenin signaling pathway. Reproduced with permission from Krishnamurthy N, Kurzrock R. Targeting the Wnt/beta-catenin Pathway in Cancer: Update on Effectors and Inhibitors. Cancer Treatment Reviews. 2018, 62, 50–60 [56].

Wnt/β-catenin is the most commonly overexpressed pathway leading to transcriptional factor activation responsible for the stimulation of epithelial to mesenchymal cell (EMT) transitions in CSCs. Wnt signaling is also dysregulated in both canonical and non-canonical molecules on TNBC [47]. To the best of our knowledge, there are 19 human Wnts and 10 Frizzled (FZD) receptors and coreceptors [47,48]. Wnt ligands (WNT5A, WNT11, and WNT3A) are pertinent in promoting migration and invasion [49]. FZD6 receptor is the most important representative in TNBC due to its capacity to produce metastasis by increasing the motility characteristics of the malignant cells in TNBC [50]. Some novel drugs target Frizzled receptors, for example, OMP-18R5 an antibody targeting Frizzled receptors diminishes proliferation of tumor cells in the lung, breast, colon, and pancreatic tumors [48]. (Figure 2) Additionally, overexpression and accumulation of β-catenin protein stimulates cell migration consequently leading to resistance in TNBC cells [47]. Wnt inhibitors and modulators can eradicate CSC clonal cells and drug-resistant cells [51], but we need to determine their safety in maintenance of tissue homeostasis and repair. The activation of Wnt/β signaling pathway is correlated to diminished clinical outcome in TNBC [52], as it presents the threat of lung and brain metastasis [53]. To date, scientists believe that pluripotent CSCs play key role in the formation of the primary malignant solid tumors. These CSCs are also responsible for the formation of drug resistance proteins in breast cancer, and are strongly implicated in metastasis [54,55].

### 2.4. Poly (ADP-Ribose) Polymerase (PARP) Inhibitors

The polyadenosine diphosphate-ribose polymerase also called poly (ADP-ribose) polymerase (PARP) is a superfamily of 18 proteins that effect all the molecular events that leads to recovery of the cells from DNA damage (participate in DNA base excision repair), gene transcription, apoptosis and genomic stability [57].

**Figure 3:**
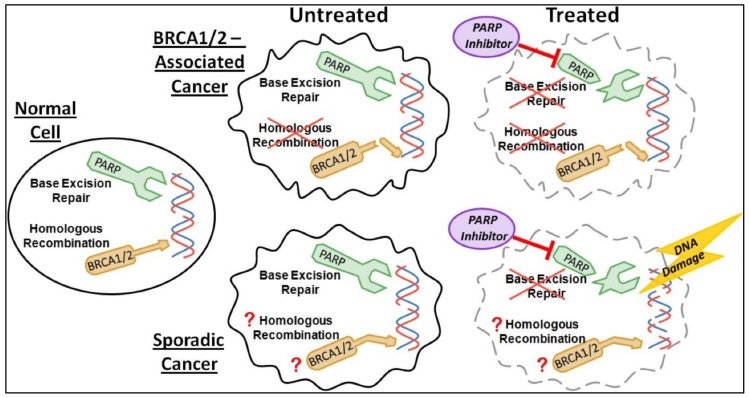
Poly (ADP-ribose) polymerase (PARP) inhibitor treatment of BRCA-1/2-associated and sporadic cancers. Reproduced with permission from Leif W. Ellisen. PARP Inhibitors in Cancer Therapy: Promise, Progress, and Puzzles. Cancer Cell. 2011, 19(2), 165–167 [66].

Roughly, 70% breast cancers evolving in BRCA1 mutation carriers while 23% of breast cancers evolving in BRCA2 carriers, express a triple negative phenotype [58]. Therefore, PARP inhibitors are considered perhaps the most important therapeutic drugs under investigation for the BRCA-1 and BRCA-2 mutations as well as against TNBC. PARP expression in TNBCs is a consequence of exposure to chemotherapy. PARP-1 and PARP-2 proteins are induced by DNA strand breaks and are associated in DNA repair processes. PARP synthesized ADP-ribose polymer drives both BER (excision repair pathway) and single-strand break repair (SSBR) pathways [59] (Figure 3).

PARP activity, when suppressed, inhibits the ADP-ribose complex formation, so PARP-dependent DNA-damage repair complexes such as DNA polymerase ε [60] cannot be efficient for repairing DNA-damage [61]. Trapped PARP-DNA complexes are extremely cytotoxic exhibiting high anti-proliferative activity (and therefore anticancer activity) [62]. Furthermore, Olaparib (AZD-2281) and Veliparib (ABT-888) (both are PARP inhibitors) also differed markedly with respect to their catalytic inhibitory propensities. Thus, the clinical as well as experimental results of each PARP inhibitor also varies with respect to inhibition [63,64]. Since PARP inhibitors are different with respect to trapping PARP-DNA complexes [62,65], differences can be seen while comparing the two (Olaparib and Velipamib) with Velipamib the less dominant drug repressor of PARP1 and PARP2 than Olaparib [62].

### 2.5. Mammalian Target of Rapamycin (MTOR) Inhibitors

The erroneous regulation of mammalian target of rapamycin (mTOR) signaling, especially Phosphoinositide- 3 kinase (PI3K)/Akt/mTOR pathway has a direct relationship with malignancy [67]. The mTor pathway is transformed in TNBC patients, thus is responsible for poor prognosis (aggressive and tissue invasion) [68].

**Figure 4:**
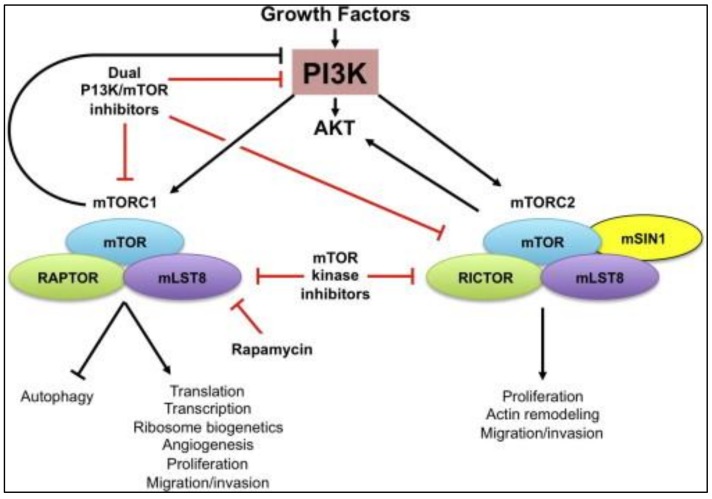
Mammalian target of rapamycin (mTOR) signaling pathway. mTOR is a subunit of two distinct multi-protein complexes, mTORC1 and mTORC2. Both mTORC1 and mTORC2 can be activated in response to growth-factors stimulation, whereas mTORC2 is a major kinase that phosphorylates and activates Akt. The importance of mTORC1 and mTORC2 in regulation of multiple cell functions vital for development of cancer and their strong interaction with oncogenic pathways make mTOR an attractive target for therapeutic intervention. The mechanisms of action of currently available mTOR inhibitors are shown. Reproduced with permission from Zaytseva YY, Valentino JD, Gulhati P, Evers BM. mTOR inhibitors in cancer therapy. Cancer Letters. 2012, 319, 1–7 [68].

Phosphorylation reactions, stimulated due to the PI3K/Akt/(mTOR), are responsible for cancer cell growth, cell proliferation and angiogenesis [69]. Moreover, overexpression of Akt, a protein kinase, is also correlated with tumor metastasis and invasion [68] The downstream signaling cascade of the PI3K/Akt pathway is mTOR that is present in two functionally different complexes (mTORC1 and mTORC2). The mTORC1 pathway promotes mRNA translocation as well as phosphorylates a wide range of of substrates that accompany many anabolic processes [68] (Figure 4).

There are 6 classes of PI3K/AKT/mTOR network inhibitors: 1. Pan-class I (PI3K blocker) 2. Isoform-selective (PI3K blocker) 3. Rapamycin analogs (Rapalogs: Everolimus, Temsirolimus, Deforolimus), 4. Active-site (mTOR blocker), 5. Pan-PI3K/mTOR blocker, and 6. AKT blockers [68]. Additionally, mTOR and one PI3K isoform, can be targeted simultaneously to increase the efficiency as compared to single PI3K inhibition [68].

### 2.6. Epidermal Growth Factor Receptor (EGFR)

**Figure 5:**
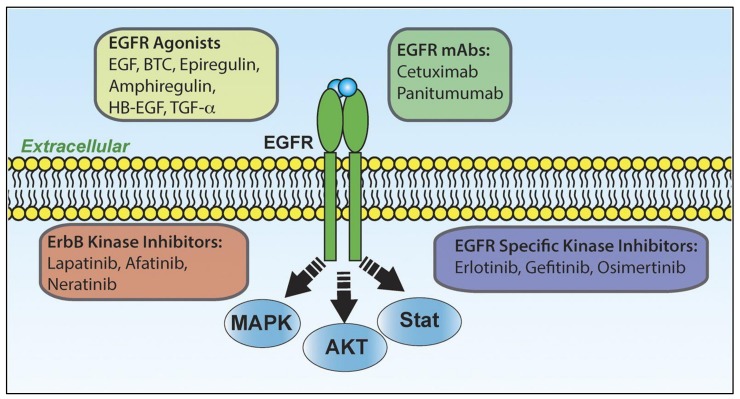
A schematic representation of the activators, inhibitors and outcomes of epidermal growth factor receptor (EGFR) signaling. EGFR is part of the four-member ErbB superfamily (ErbB1–4). These receptors form several different homo- and heterodimers (here we only depict the EGFR homodimer). EGFR is capable of binding several different extracellular ligands that agonize the receptor leading to activation of several downstream signaling events including, but no limited to those listed. Several therapeutics have been developed to antagonize EGFR including monoclonal antibodies (mAbs) that block ligand binding as well as several different kinase inhibitors. In addition to EGFR, some of these kinase inhibitors also target other ErbB receptors, supporting their use in human epidermal growth factor receptor-2 (Her2)-amplified breast cancer (BC). All of the listed therapies are Food and Drug Administration (FDA) approved for various cancers with the exception of Neratinib. Reproduced with permission from Ali R and Wendt MK. The paradoxical functions of EGFR during breast cancer progression. Signal Transduction and Targeted Therapy 2017, 2(16042), 1–7 [81].

Receptor tyrosine kinase (RTK) targets such as epidermal growth factor receptor (EGFR) expression are reported in 89% of TNBC cases, and hence considered to be a valid therapeutic target, especially for BL2-subtype tumors that are augmented in EGFR gene expression [70]. Activation of this gene stimulates primary tumorigenesis as well as metastasis. Gefitinib (EGFR inhibitor) reduces cancer cell multiplication and enhances the cytotoxicities of carboplatin and docetaxel (Figure 5) [70,71]. There are a number of different kinds of EGFR inhibitors trialed against TNBC such as the tyrosine kinase inhibitors (TKIs)-erlotinib and lapatinib along with the monoclonal antibodies (mAbs) such as cetuximab and panitumumab [72,73,74,75]. The reports of failures of EGFR-TKIs and mAbs, however, inspired combination therapy that includes mAbs and chemotherapeutics that proved to be a more efficacious. As an example, cetuximab and carboplatin as well as Cetuximab and cisplatin in advanced TNBC patients, showed double the efficiency of therapeutic response [76,77]. Moreover, the tri-inhibitors together namely, gefitinib, carboplatin, and docetaxel synergistically increased the cytotoxicity of TNBC cells [78].

Another drug, cannabidiol caused inhibition of breast cancer metastasis by blocking the EGF/EGFR signaling pathways and alteration of the tumor milieu [79]. Hence, cannabidiol could potentially be efficient therapeutic strategy for highly aggressive TNBC [80].

### 2.7. Rapalogs

Rapamycin as well as paclitaxel both affect the PI3K/AKT/mTOR pathway, thus playing an important role in therapeutics of TNBC. The mTOR antibodies coupled with EGFR inhibitors are more effective as compared to anti-mTOR alone, even though there is no known evidence about the synergy between anti-EGFR as well as mTOR inhibitors [81]. There is a report in which novel oral AKT inhibitor Ipatasertip helps in progression-free survival alongwith PI3K/AKT pathway activation in TNBC patients. But still, there is an urgent need to synthesize novel inhibitors that can target PI3K/Akt/mTOR pathway for TNBC therapeutics [82].

### 2.8. TGF-β Signaling Pathway

TGF-β1 belongs to the TGF-β superfamily of cytokines encoding *TGF-β1* gene. Human platelets, which are a 25kDa cytoplasmic fragments have an important role in wound healing as well as in regulation of the immune system. Thus, it inhibits the secretion and activities of different cytokines such as IFN-gamma, TNF-alpha, and IL-2. TGF-beta 1 has an important activity in breast cancer stem cells, as they express TGF-β1 *and the* TGF-β1 receptor exponentially [83,84]. TGF-β inhibitors can inhibit the growth and multiplication of chemotherapy-resistant tumor-initiating cells (TIC) in vivo [84] forming the basis for combinatorial chemotherapy for patients suffering from TNBC. TGF-β stimulates an epithelial-to-mesenchymal transition (EMT) within mammary cells, leading to an exhibition of tumor-like properties. It is possible to reverse EMT via TGFBR1/2 inhibitors while stimulating mesenchymal-to-epithelial (MET) differentiation inside mammary epithelial cells [85]. TGF-β is frequently found overexpressed in the TNBC tumor microenvironment, especially in tumor cells, or by tumor-associated immune and stromal cells. These cells also generate SMAD2/3 and SMAD4, thus leading to metastasis and angiogenesis. This indicates that the TGF-β inhibitors play an important role in patients with metastasis [85]. 

### 2.9. CSPG4 Protein Signaling Pathway

The CSPG4, which is also known as non-glial antigen or its also known as melanoma chondroitin sulfate proteoglycan, is a cell-surface proteoglycan exhibited by basal breast carcinoma cells. Inhibition of CSPG4 is therapeutically effective for breast cancer therapy. This protein leads to the dissemination of the endothelial basement membrane protein, thus stabilizing the cell-substratum interaction, which is in similitude to the effects that occur in TNBC. CSPG4 monoclonal antibodies can cause a blockade of migratory, mitogenic and survival signalling pathways in tumor cells, making CSPG4 a new TNBC target [86]. Moreover, there is overexpression of CSPG4 in TNBC cell types, resulting in inhibition of TNBC cells when CSPG4 was targeted in such cells [87].

### 2.10. Cancer Stem Cells (CSCs) and Autophagy

As mentioned, numerous biochemical pathways in TNBC are relevant to cancer stem cells (CSCs), thus, efforts are ruining into mAbs, dendritic cells (DC) and pluripotent cells cancer vaccines as well as adoptive immunotherapy [88].

TNBC cancer stem cells (CSC) feature enhanced proliferative capacity, refractory treatment which leads to recurrence and metastasis (CD-24, CD-44) [89]. Several biomarkers have been designed to detect CSCs. However, most biomarkers are also shared by normal stem cells, and therefore these biomarkers become to unspecific molecules leading to side effects. Chemo-resistance is present in TNBC stem cells, and they are the ‘generals’ that lead the battle in tumor micro-environments riddled with hypoxia [90]. Hypoxia is responsible for increasing chemo-resistance of autophagic TNBC stem cells. Blocking the autophagic cascade network can increase chemo-response [91].

Autophagy is required for cancer stem cells and autophagy processes helps in the maintenance of cellular homeostasis and, therefore, represents a survival pathway in cells. Unfortunately, cancer cells can regulate the autophagy pathway to develop resistance to chemotherapy. Therefore, molecular inhibition of the malignant autophagic pathway could reverse resistance to chemotherapy [91]. More research needs to be done regarding abnormal stem cells autophagy mechanism, since it may harbor the key to get a definitive cure not only against TNBC but against many types of cancer.

## 3. Strategies for TNBC Therapeutics

Despite the discovery of new metabolic and biochemical pathways within tumor microenvironments, scientists and physicians continue to develop strategies to block network routes and signals of neovascularization, metastasis, activating apoptosis, and “awakening” the immune response [92]. This effort is made challenging by the dynamic and chaotic molecular configuration of tumors, allowing tumors to recruit stromal cells, use valuable resources such as organic metals, vitamins, and create their own blood supply using aberrant signaling. The standard approach has been to use cytotoxic therapeutics, the chemotherapies that since the 1970s have been assisting oncology patients; however, these approaches lack the desired selectivity.

Promising unconventional therapeutics are based on materials systems including nanopolymers, liposomal drug delivery and nanostructured materials [93]. In 1995, the FDA approved Doxil^®^, the first liposomal nanodrug part of a novel chemotherapy superior to the conventional [94] and a year later the FDA also approved Feridex^®^, nanoparticles for magnetic resonance imaging [95]. In this context, conventional chemotherapy and diagnostics are embracing the field of nanotechnology, promising to provide valuable specificity to the treatment of cancer.

### 3.1. Conventional Therapeutics

#### 3.1.1. Neoadjuvant Therapy

Currently, chemo-resistance is a significant problem for oncologists, with up to 90% of drug failures in metastatic cancers [96]. TNBC patients initially respond to neoadjuvant treatments. Unfortunately, there is a possibility of relapse in patients in the first 5 years in comparison with other cancer subtypes [97]. Nevertheless, neoadjuvant chemotherapy is the TNBC gold standard treatment [98]. It is important to be diligent in TNBC patients, however, with the proper choice of drugs improving prognosis [99]. Additionally, neoadjuvant anthracycline–cyclophosphamide (AC-scheme) chemotherapy appears to be establishing efficacy, although recently there have been reports on resistance developed for these drugs [100]. Scheme AC in the presence of BRCA mutations has a pathological complete response (pCR) rate of 27–30% [101] and consists of: Doxorubicin and Cyclophosphamide for 4 weeks followed by Paclitaxel for 12 weeks. The prognosis may be improved to 61% if they associate drugs such as Cisplatin [102]. Other drugs can be used as Carboplatin (CALGB40603 study) or Abraxane (Nab-Paclitaxel nanoparticles) or immunotherapy using Bevacizumab [103,104]. On the other hand, after the treatment, the monitoring of the disease should be evaluated by imaging techniques like MRI which is the most sensitive imaging method for measuring TNBC neoadjuvant response-treatment [105]. Precision medicine strategies identify strategic biomarkers in each oncological patient, providing a more effective and selective chemotherapy regimen [106]. Within the margins of personalized medicine, medical research suggests that for TNBC treatment, one of the most useful molecular targets is EGFR since it is positively expressed (around 60%) in TNBC [107].

Neoadjuvant therapy improves the response rate in patients with TNBC compared to adjuvant therapy [101,108], as the effectiveness of cisplatin in TNBC is observed in preoperative phase II studies where BRCA-1 expression is deficient. [109,110,111]. However, neoadjuvant systemic therapies should be individualized because tumors with a BRCA-1 mutation are basal, but not all basal cancers express BRCA-1 mutation. Moreover, cisplatin and bevacizumab, the latest as a molecular target of VEGF, have shown to be efficient drugs in neoadjuvant therapy against TNBC [101,112] as researchers point out in different meta-analysis as *E2100, AVADO and RIBBON-1* [113]. Taxane-resistance of malignant cells expressing BRCA-1 mutation is reported in in vivo studies [114], but a clinical trial called “*CALGB 9344/INT1048”* concluded that the use of paclitaxel reduces cancer recurrence in 17% as well as a terminal clinical prognosis in 18% of TNBC patients [115].

#### 3.1.2. Adjuvant Therapy

Adjuvant therapy is also a critical strategy to avoid the risk of metastases with concomitant rapid progression and tumor recurrence activity [116]. The MA5 study showed anthracycline-based drugs were not effective for treatment when BRCA-1 is expressed in TNBC [117,118], while other studies show anthracyclines had encouraging results as adjuvant therapeutics [119]. The decision whether or not to carry out adjuvant therapy must be evaluated for each patient by means of rigorous analysis of clinical-histopathological staging-conditions and an adequate categorization of genomic and proteomic profile.

The ability of TNBC to produce metastasis has been mentioned above, and shorter survival time has been correlated with the presence of extensive tumor stroma. Therefore, it is crucial to study the chemotherapy regimen in a palliative state because it is key for the clinician to understand which drug is more effective. Many clinical trials are being done to evaluate the best treatment for TNBC, for example comparing carboplatin efficacy versus docetaxel for metastatic TNBC [120]. Although different doses of taxane have been used in metastatic breast cancer (MBC), there is no evidence indicating good efficacy in TNBC. For advanced stages (III C) when anthracycline-taxane scheme resistance is documented, Xeloda^TM^ (Capecitabine) combined with Taxotere^TM^ (Docetaxel) is administered intravenously. (Table 1) Another combination that has shown utility is Ixempra^TM^ (Ixabepilone) plus Capecitabine, although Ixempra^TM^ can be used as monotherapy at the same dose. (Table 1).

#### 3.1.3. Surgery

Many studies have been performed to determine the prognostic effects of mastectomy over lumpectomy [110]. In TNBC, the surgical treatment of choice is the preservation of the breast; this is because the choice of surgical treatment does not improve the prognosis or the local tumor recurrence, so patients remain appropriate candidates for breast conservation [122]. A lumpectomy followed by radiation therapy could be an option (National Comprehensive Cancer Network guidelines). However, in TNBC, the gold standard is neoadjuvant therapy and is preferred before surgery.

#### 3.1.4. Radiotherapy

Similar to conservative breast surgery, radiotherapy is part of the treatment regimen for TNBC, albeit with some controversy [123]. However, evidence points out TNBC-BRCA-1 aberrant expression is highly radiosensitive [124]. TNBC is considered a pathological entity susceptible to radiotherapy. But unfortunately, like the guidelines in the pharmacological treatment, the use of RT in TNBC does not have treatment guides [125,126].

### 3.2. Advanced Therapeutics

Chemoresistance is a significant problem in metastatic cancer [127]. Even though chemotherapy has reached a milestone in the treatment strategies [128], there is a need to reduce the side-effects of all the therapeutic regimens [129]. Moreover, non-steroidal anti-cancer drugs also possess many side-effects and they also exhibit severe toxicity towards normal cells apart from cancer cells [130].

There are two main types of strategy for targeting therapeutics to tumor sites:

(1) A passive transport process called “enhanced permeability and retention” (EPR) in which peripheral blood vessels to the tumor have leaky vasculature that increases nanoparticle permeability. However, the disadvantage of EPR is that not all tumors possess leaky vasulature. Therefore an adequate analysis of TNBC tumor biomarkers is required to load the nanoparticles with a ligand specialized in the search for receptors overexpressed like CXCR4 (folic acid receptor) [131].
(2) Another approach used by researchers is active transport that is governed by using biomarkers miRNA (microRNA), proteins, antibodies, as well as therapeutic biomolecules such as siRNA and aptamers, discussed below.

#### 3.2.1. miRNA

The importance of microRNA (miRNA/miR) related with cancer treatment has recently increased due to their potential as diagnostic biomarkers [108]. In TNBC, miRNA558 is the one that is overexpressed [132]. Moreover, several TNBC miRNAs were found in a metanalysis [133]. Detecting miRNAs promise to be part of the arsenal of oncological studies that will be available in hospitals to provide better diagnosis and prognosis as powerful biomarkers. The most important study that is considered to be the first of its kind, focused on primary TNBC as well as normal tissues, was the microRNA profiling that discovered almost 116 microRNAs that have been deregulated. Among them, miR-106b, the cluster miR-17/92, miR-200 family (miR-200a, miR-200b and miR-200c), miR-21 and miR-155 were the highly expressed ones [134]. Furthermore, a second module of mRNA profiling of TNBC linked to lymph node metastasis showed 6 miRNAs that was expressed differentially in the lymph node tissues, namely, miR-424, iR-125a-5P, miR-627, miR-579, let-7g, miR-101 [134].

#### 3.2.2. siRNA

Since the discovery of the *Caenorhabditis elegans* plant’s properties, siRNA has generated a revolution in the treatment of diseases, with siRNA used to switch off or change the tumor genes responsible for drug resistance, and in this way increase the efficacy in the treatments [135,136]. The siRNA screens was performed for plethora of genes in TNBC cell lines and it was found that RSK2 [137]. non-SMC condensin I complex subunit D2 (NCAPD2) [138], Gpx1 (Glutathione peroxidase-1) [139], all of them act as promising therapeutic targets for the TNBC treatment. The siRNAs that have already been used in animal models to fight TNBC can be loaded in nanoparticles (non-viral) and viral capsids or supramolecular complexes, providing gene silencing for proteins that reflect poor prognosis in oncological medical practice viability [140].

Exosomes also play an important role in delivering siRNA for the suppression of metastasis of TNBC after operative surgery. Cationic BSA coupled with siS100A4 as well as exosome membrane covered nanoparticles helps in delivery of SiRNA to inhibit the growth of malignant TNBC metastasis [141].

#### 3.2.3. Aptamers

Aptamers are molecules made up of nucleotides, generally in a range of 50 DNA or RNA bases, that are evolved to bind to specific molecular targets. Their small size makes them suitable to reach molecular targets, therapeutic targets, protein complexes and cancer cells [142]. Engineering of aptamers is based on a technique called systematic evolution of ligands by exponential enrichment (SELEX). Aptamers are easier and cheaper to produce than mAbs, but the degradation of aptamers in the bloodstream is a clear disadvantage [143]. However, scientists are developing “mirror aptamers” (Spiegelmers) enantiomers highly resistant to enzymatic degradation through synthetic biology to avoid aptamer degradation by nucleases [144]. SELEX consists in the amplification of RNA (oligoribonucleotides) or DNA (oligonucleotides) [145] using PCR (polymerase chain reaction) to subsequently incubate them with molecular targets (cells, protein complexes, etc.). After five rounds the maximum molecular affinity is generally obtained [146]. Various aptamers have been developed for therapy in TNBC; for example, the aptamer 5TR1 pursues the molecular target MUC1 which is a tumor protein in MDA-MB-231, the researchers have conjugated 5TR1 to Doxorubicin to make it more specific and avoid the known side effects such as cardiotoxicity [147]. He et al. have reported aptamer-drug conjugate (ApDC), AS1411-triptolide conjugate (ATC) for TNBC therapeutics with higher efficacies [148]. Nanomedicine is advancing rapidly, and now researchers have focused on combining all the technologies mentioned above (siRNA, miRNA, aptamers) by loading them into nanoparticles that pursue the CD-44 receptor characteristic of TNBC pluripotent cells [149].

#### 3.2.4. Nanomedicine: Armadas for TNBC Therapy

There are a number of clinically approved nanomedicines used in hospitals around the world, e.g., liposomal doxorubicin (DoxilTM) [150], albumin-bound paclitaxel or Nab-Paclitaxel (AbraxaneTM) [151] and polyethylene glycol (PEG-1) Asparaginase (OncasparTM) [152]. Additionally, many nanomaterials have been studied with functions that includes: delivering drugs, aptamers or microRNA capable of inducing gene or immunological therapy [153]. Some examples of these delivery vehicles include micelles [154], luminescent carbon nanodots [155], nanodiamonds (NDs) [156], carbon nanotubes (CNTs) [157,158], Au-nano matryoshkas [159] as well as SPIONs (superparamagnetic iron oxide nanoparticles (NPs)) [160,161] etc. It is imperative to fabricate nanoparticles with the correct properties for cancer therapeutics. These properties are dependent on the method of synthesis and characterization employed. Obviously, nanomaterials for biomedical applications must be non-toxic and biocompatible. It is also necessary that the synthesis and purification methods for nanoparticles be reproducible [162], providing uniformity in size and shape; characterization that can be verified through microscopy tools. Applications in nanomedicine likewise require nanoparticles that are easy to metabolize by the human body, or be eliminated via renal or hepatobiliary clearance [163]. Nanomedicine provides a potential pathway to solve many of the problems of cytotoxicity and the lack of tumor specificity of conventional chemotherapies. NPs can also minimize off-target effects. As an example, lonidamine is an inhibitor of aerobic glycolysis but has failed in clinical trials due to its intense hepatotoxic activity. Recently, however, NPs have been developed that incorporate lonidamine together with a monoclonal antibody, providing greater selectivity for malignant cells than for the healthy cells and reducing undesired systemic side effects [164]. Because there are several numbers of tumor markers different from the PR, ER and Her-2 neu hormone receptors expressed in TNBC, the NPs can be of help to achieve greater specificity and efficiency in the treatment, being able to pursue other molecular objectives [165]. Gold nanorods have been used to carry out siRNA against MDA-MB-231 cells (TNBC), so researchers believe they could be useful for reducing tumoral activity [166]. Therefore, it is now accepted that nanotechnologies are now part of the oncologist’s therapeutic arsenal including breast cancer [167]. Nanomedicine has promise for improving the specificity with which drugs and other molecules are transported using nanoparticles that maximize the therapeutic effect and decrease the systemic toxicity of conventional chemotherapies. These nanocarriers must first have an adequate safety profile, the parameters of which must be determined [168]. Therefore, understanding nano-pharmacokinetics and nanotoxicology is mandatory [168]. Functionalized nanoparticles for cancer therapeutics and diagnosis can be fabricated from diverse materials such as gold, silver [169], diamonds [170], copper [171], among others. These materials are used due to their low cytotoxicity, for example, gold nanoparticles are not cytotoxic making them suitable candidates for nanomedicine [172]. Currently, nanodrugs as DaunoXome^®^ or Doxil^®^ are currently being used in oncology, more research is needed to switch onto the next pillar-Nanomedicine. Immunotherapy is promising to reduce tumoral recurrence, improving conventional treatment and reducing side-effects. However, more studies need to be done. An important challenge in nanomedicine is how to engineer nanoparticles to evade the immune system. The researchers responded by adding liposome layers, polyethylene glycol PEG coatings that reduce the recognition of macrophages (evacuation of the reticuloendothelial system), which increases the bioavailability and half-life of the drug [172]. Another useful coating could be SDS, CTAB or tween 20 [173]. Even more promising, but not unrealistic, is the concept “Theranostics,” in which nanoparticles can diagnose and treat at the same time. The nanoparticles can be used to deliver drugs and generate real-time images,” [174]. Current intrahospital photodynamic therapy (PDT) consists of administering porphyrins and phthalocyanines that have an affinity for malignant cells; then, a laser can stimulate the structure and cause the release of reactive oxygen species (ROS). Nanotechnology takes PDT to a different, enhanced level. PDT based on nanoparticles exploits a photosensitizing agent creating ROS and apoptosis avoiding healthy tissue damage [175]. Many other studies were carried out finding utility and efficacy in the joint use of NPs that act as a photosensitizer to produce PDT [176,177].

There are many different kinds of nanoparticles exploited for cancer therapy which are as follows:

**(i) Quantum Dots (QD)**

Quantum dots (QDs) were discovered in 1982,and are semiconducting nanocrystals that have superior light absorbance and high fluorescence intensity [178]. QD-based nanotechnology possesses wider applications in cancer molecule imaging and quantitative detection [179]. Many studies using QD technology could substitute immunohistochemistry (IHC) [180], because of its better fluorescent signaling, performing even more accurate quantitative analyses for evaluating prognosis in TNBC [181]. QDs demonstrate results of molecularly directed images, as well as better quantitative detection of cancer molecules like Ki67 and EGFR, expressed on TNBC [182].

**(ii) Fluorescent nano-diamonds (FNDs)**

Current nuclear medicine uses radioisotopes such as strontium-89, iodine-131, samarium-183 and technetium-99. However, nanotechnology proposes the use of non-radioactive materials with improved sensitivity and specificity. In this technological revolution, we can also find materials such as fluorescent nano-diamonds (FNDs). Fluorescent nano-diamonds are biocompatible nanomaterials often used in MDA-MB-231 theranostics [156,183].

**(iii) Nano-matryoshkas**

Another singular design has been developed as thermal therapeutic, imaging and drug-delivery nanoparticles. Nano-matryoskka, referred to as a multi-layer nanoparticle reminds of the Russian doll which can contain many other dolls inside, the application of the hollow nanoparticles capable of delivering multiple drug loads contained in multilayers that can be designed with different materials as suggest an MDA-MB-231 murine xenograft study [184].

**(iv) Silver nanoparticles (AgNPs)**

Silver Nps (AgNP) are another example of Nps that can act against tumor cells in TNBC that can induce DNA damage as in vivo studies suggest. Silver nanoparticles help in reduction of TNBC growth and augments radiation therapy [172]. The mechanism of action is physical, it has not been specifically established. One possibility is that the reaction of silver in the cellular microenvironment will lead to the release of reactive oxygen species.

**(v) Gold nanoparticles**

Gold nanoparticles are photothermally tunable since they exhibit plasmonic behavior when exposed to light, a unique property of matter at the nanoscale. These plasmonic NPs are useful for producing heat and bringing apoptosis through hyperthermia [185]. Taking advantage of the near-infrared (NIR) wavelength for medical applications, hyperthermia can kill cells due to reasonably efficient tissue penetration of NIR radiation. [186]. It is effective when combined with radiotherapy and chemotherapy [187]. Hyperthermia therapy or photothermal therapy continues to be the subject of research in nanomedicine, because at less than 100 nm the electromagnetic properties of materials allow heat generation, prompting innovative treatments and diagnostics (theranostics) [188].

**(vi) SPIONs (superparamagnetic iron oxide nanoparticles) and core-shell nanoparticles**

The ability of iron oxide NPs to produce strong contrast images in RMI in T1(longitudinal relaxation—spin-lattice) and T2 (transversal relaxation—spin-spin) has given them a place in the theranostics of cancer [189,190]. This novel imaging system using iron oxide nanoparticles (IONP) has been used in several xenograft model [191,192], for MRI diagnostic in TNBC [188]. SPIONs have higher magnetic properties than paramagnetic materials due to their ability to spin alignment to an external magnetic field, SPIONs can generate heat inside the tumors producing apoptosis by hyperthermia [193]. SPIONs’ core-shell are formed by layers: an iron oxide core and a therapeutic biocompatible coating [185] which can reduce toxic side effects [194]. Hayashi et al. [195] have shown the advantages of using SPION intravenously for cancer theranostics. Also, SPIONs core-shell hyperthermia properties, have been the hallmark of this design. Researchers are using lasers [196], ultrasound [197], radio frequencies [198] or alternating magnetic fields [199] to generate apoptosis. Moreover, SPIONs are also useful to deliver anti-cancer drugs such as gambogic acid (GA) in TNBC. Sang et al. developed a GA drug nanoconjugate with an outer layer made up of mono-aminated poly (ethylene glycol)-grafted hyaluronic acid that can specifically target CD44 receptors on TNBC (Hyaluronic acid has higher affinity to bind CD44 on TNBC); the middle layer comprises disulfide-linked hexadecanol (Hex) as well as chitosan oligosaccharide (CSO) that controls the drug release, while the core layer is made up of SPIONs attached to GA that can increase the enhanced permeation and retention effect due to magnetic focusing. This complex of mPEG-HA/CSO-SS-Hex/SPION/GA nanosystem led to efficient delivery of the drug using magnetic guidance to focus in TNBC microenvironment [200].

**(vii) Nanocomposites and their advantages over core-shell nanoparticles**

The core-shell modality, however, has great challenges, including the negative polarity and the amphipathic characteristic that makes them an easy target for the immune system. Alternatives in nanoengineering are the creation of nanocomposites [201] which consist of biphasic or multiphase materials, respecting the condition that at least one dimension of the material has less than 100 nm [202]. The improved optoelectronic properties allow nanocomposites to be useful candidates for drug delivery, food packaging [203], sensing devices and their antimicrobial properties are currently being studied [204]. Nanocomposites’ advantages over core shell design relies on colloidal easy synthesis and reproducibility, because different matrices of materials can be fused regardless of their polymeric or porous structure [205]. Administration as colloids would guarantee an adequate renal clearance if the assemblies are either smaller than ~6 nm or can degrade into components of this size. Nevertheless, a clear disadvantage in contrast with core-shell design is found in the largest size (100 nm) which is characteristic of nanostructured materials [206].

**(viii) Polymeric nanoparticles**

In cancer therapeutics the major drawback of many nanocarriers as well as synaphic moieties is that they bind non-specifically to many cellular as well as extracellular matrices, thus creating a barrier for effective drug delivery. There are recent reports in which material scientists have utilized a nanoparticle drug conjugate formulation in such a way that there is very less interaction with the blood or other tissue sections which are named DART nanoparticles (poly(lactic-co-glycolic acid) (PLGA)-polyethylene glycol (PEG)–ITEM4 nanoparticles). ITEM4 or Fn14 monoclonal antibody binds specifically human as well as murine Fn14 extracellular domain. Paclitaxel loaded-DART nanoparticles is an FDA-approved nanoformulation for TNBC models as well as an intracranial model thus indicating that there is TNBC growth which is followed by metastatic propagation to the brain [207]. Furthermore, Xu et al. developed Hyaluronic acid-coated pH sensitive poly (Beta-amino ester) nanoparticles for the delivery of both embelin (anti-cancer drug) as well as pTRAIL (tumor necrosis factor-related apoptosis-inducing ligand (TRAIL) plasmid for anti-TNBC efficacy [208]. All the above nanotechnologies for health care are presented in Table 2.

## 4. Immunotherapy

**Figure 6:**
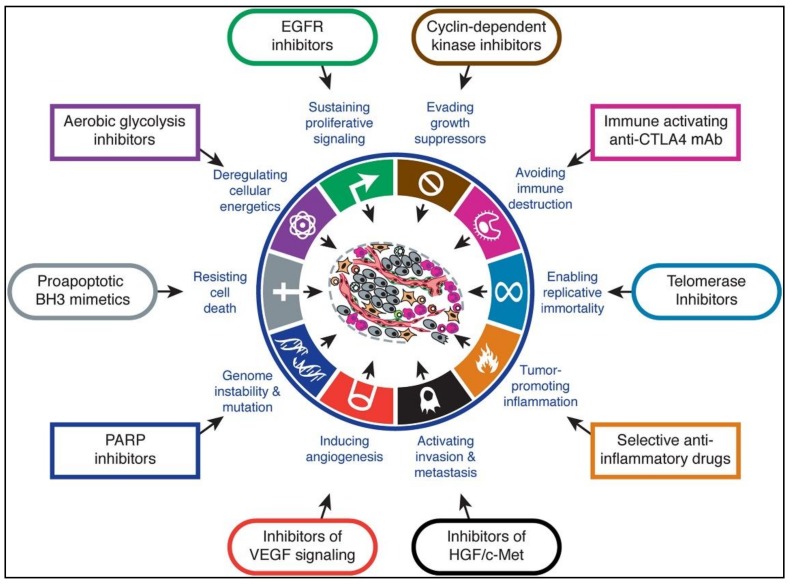
Therapeutic targeting of the hallmarks of cancer drugs that interfere with each of the acquired capabilities necessary for tumor growth and progression have been developed and are in clinical trials or in some cases approved for clinical use in treating certain forms of human cancer. Additionally, the investigational drugs are being developed to target each of the enabling characteristics and emerging hallmarks depicted in Figure 3, which also hold promise as cancer therapeutics. The drugs listed are but illustrative examples; there is a deep pipeline of candidate drugs with different molecular targets and modes of action in development for most of these hallmarks. Reproduced with permission from Hanahan & Weinberg. Hallmarks of Cancer: The Next Generation, Cell 144, 4 March 2011 Elsevier Inc. 2011,144(5), 646–674 (215).

Since they were described, the criteria of Hanahan and Weinberg have undergone constant modifications, adding more functions and properties that confer to the cancer cells the capacity of proliferation and invasion to other tissues in addition to having an improved machine for its survival. The hallmarks are described in Figure 6 [215].

Researchers have focused their efforts on making immunological-portraits of the tumor microenvironment in TNBC [216] by using computational tools such as Cell-type Identification by Estimating Relative Subsets of RNA Transcripts (CIBERSORT) [217] and Estimation of Stromal and Immune cells in Malignant Tumors Using Expression data (ESTIMATE) [218] that allow the evaluation of gene expression in solid tumors, including those for immune response interest.Significantly, they confirmed that TNBC has the worst outcome because of the metastasis-promoting genes having higher expression as well as the depressed expression of metastasis-inhibiting genes. Finally, to make things more complicated, in the same study, they also documented that TP-53 mutations are more common in TNBC leading to tumor immunosuppression [216]. Altogether, this data allows us to advance to the objective of seeking therapeutic targets and understanding TNBC microenvironment.

But even when transcriptomics and genomics generate insights on TNBC, it is difficult to approach therapeutics in such an aberrant environment. Within the TNBC tumor microenvironment, there are latent T and B lymphocytes, antigen-presenting cells (APCs) which for some reason do not respond to the threat posed by tumor cells. Recently, strategies have been implemented to “wake up” these cells from their quiescent state and initiate a response that slows the progression of the tumor [219,220]. It is assumed that cytotoxic T-lymphocyte antigen-4 (CTLA-4) helps in the downregulation of the immune response [221]. So, the current efforts are directed to activate immune system response by using CTLA-4 inhibitors such as ipilimumab. The challenge, however, is that they activate T-cells in an aggressive way, generating different systemic adverse effects [222].

Ipilimumab (anti-CTLA-4), and very recently pembrolizumab (anti-PD-1) and atezolizumab have been approved by the FDA in patients with overexpression of PD-L1. The Nobel Prize in Medicine (2018) was given for both anti-CTLA-4 and PD-1 discoveries. The programmed cell death-1 (PD-1) signal pathway has a ligand called 1 (PD-L1) [223]; both can lead to programmed death on malignant cells [224], especially for TNBC [225]. Atezolizumab helps in the therapeutics in TNBC patients with advanced or metastatic cancer [226]. Nevertheless, in order to prevent disease progression or avoid side effects such as alopecia, thrombocytopenia, anemia and neutropenia, the FDA recommends atezolizumab dose is 840 mg intravenous (IV) infusion over 60 min, followed by 100 mg/m^2^ paclitaxel for 28-day cycle.

Two new PD-L1 inhibitors, mepolizumab and nivolumab, are being tested in an ongoing clinical trial. Unfortunately, 76% TNBC patients having PD-L1 expression have not shown therapeutic response under mepolizumab therapy [227]. Tumor-associated macrophages (TAMs) are the most efficient part of tumor immunosuppressive microenvironment that allows tumor growth as well as metastasis. Li et al. synthesized porous hollow iron oxide nanoparticle (PHNPs) and then loaded PI3K inhibitor, known as 3-methylaadenine (3-MA), which is further mannosylated to specifically target TAM. This combination of PHNP and mannosylated 3-MA synergistically activates the inflammatory factor NF-kappa B p65 of macrophages as well as helped in switching TAMs to M1-type macrophages (M1 is pro-inflammatory in nature). This resulted in activation of immune response ad inhibition of tumor growth in vivo [228].

## 5. Artificial Intelligence

At the end of the second decade of the 21st century we can see the power of artificial intelligence (AI) not as science fiction, but as a real tool in fields such as engineering, marketing, military industry and medicine [229]. In medicine, AI has an arsenal of complex statistical software models that are based on machine learning that can be used for diagnosis and therapeutics. Some examples of these techniques are computer-aided detection (CADe), case-based reasoning (CBR), osteodetect machine learning, computer-aided diagnosis (CADx), explainable artificial inteligence (XAI) and rainbow boxes [230,231]. In 2017 Seroussi et al. used XAI and CBR to create DESIREE (Decision Support and Information Management System for Breast Cancer) which can interpret and predict breast cancer disease by optimizing the treatment with metadata and confirmation from medical oncologists who validated the effectiveness of this AI in the service of oncology [232]. AI also has been implemented as a tool in breast cancer screening [233].

Metadata, also called metaheuristic data, are provided by real oncology-patient data banks, treatments and trends are analyzed qualitatively and quantitatively. It is hoped that in the future AIs can help physicians open up a faster and more accurate theranostics for building and elucidate extremely complex decision algorithm, as cancer represents it [234].

Recently, Fernández Martínez et al. [235] published the design of an AI algorithm based on machine learning that was able to help oncologists to detect different TNBC subtypes for optimizing therapeutics. In the near future, a sophisticated AI could feed back different databases such as TCGA (The Cancer Genome Atlas) [236,237] which contains SNP-based platforms, reverse-phase protein array (RPPA), DNA and RNA sequencing among other useful critical information working in conjunction with METABRIC (Molecular Taxonomy of the Breast Cancer International Consortium) [238] to study molecular heterogeneity in various molecular subtypes of breast cancer including TNBC, they could provide valuable Big Data (clinical history, transcriptomic, recurrence, prognosis, treatment, etc.) for oncologist to bring personalized medicine to these patients in a reasonable time. Unfortunately, to date there have not been many publications about this and more multidisciplinary research on AI in medicine is necessary.

## 6. Conclusions

The aberrant signaling observed in the tumor microenvironment is a consequence of the genomic instability in oncological patients. However, TNBC studies suggest that immunity in the patients is suppressed and they have the worst prognosis of all breast cancers. Therefore, it is imperative to understand the signaling phenomena in the tumor microenvironment and implement a multidisciplinary approach to diagnosis and therapy. Molecular signaling-based therapies are the basis for the comprehension of their diversity. After complete analysis of TNBC both at genetic as well as proteomic levels, this signaling assay tool has formed an important asset for clinical trial development. There are hotspot mutations or pathognomonic origins of TNBCs that need to be rectified for effective therapy. All molecular therapies can be coupled with synaptic marshaling agents such as aptamers and antibodies for increasing the efficacy of TNBC therapeutics. Moreover, there are physical therapies such as hyperthermia, photothermal and photodynamic therapies that increase the delivery of drugs in the most drug-resistant zones. This multi-pronged marshaling mechanism helps in complete eradication of TNBC.

The new discoveries in molecular biology, immunology, nanotechnology, and computer networks will allow the clinician to have more tools to make earlier and more accurate diagnoses to provide personalized treatment. Currently, efforts are focused on micro and nanofluidics to study the tumor microenvironment and understand more about the dynamic processes of cancer. Currently, nanoelectromechanical systems (NEMS) and microelectromechanical systems (MEMS) [239] are being used for tracing exosomes (cell vesicles with specific surface markers) [240] that are not present in healthy pluripotential cells, but only in the tumor.

## Figures and Tables

**Table 1:** Conventional treatment of triple negative breast cancer *.

Conventional Treatment	Drugs	Mechanism	Scheme/Dose	References
Neoadjuvant treatment Early TNBC (Gold standard) Advanced or Metastatic	Anthracyclines + Taxanes **or** Capecitabine + Taxane Ixabepilone monotherapy or Ixabepilone + Capecitabine	Cytotoxicity ** Stabilization microtubules	Doxorubicin 20 mg/m^2^ plus Cyclophospamide 600 mg/m^2^ 4 weeks followed by Paclitaxel 80 mg/m^2^ 12 weeks Capecitabine 1250 mg/m^2^ 14 days + Docetaxel 75 mg/m^2^ Ixabepilone 40 mg/m^2^ per 3 weeks	[101]
New neoadjuvant agents (BRCA mutations)	Platinums (Carboplatin) Bevacizumab, Nab-paclitaxel.	Cytotoxicity and VEGF immunotherapy	Adding up standard scheme Abraxane 125 mg/m^2^, Carboplatin AUC, Bevacizumab 10 mg/kg	CALGB 40,603 trial [104]
Adjuvant agents	Anthracyclines and Taxanes	Cytotoxicity	Cyclophosphamide 600 mg/m^2^ + Doxorubicin 20 mg/m^2^ + Docetaxel 75 mg/m^2^ for q3 weeks 6 cycles.	[121]
Surgery: TNBC, surgical treatment is breast preservation.				
Radiotherapy: radiation therapy (RT) is often given combined or after chemotherapy. RT also could be useful after surgery. Probably benefits in BRCA mutations.				

* The conventional treatment presently prescribed in hospitals for TNBC (Triple Negative Breast Cancer). It depends pertinently on the clinical stage of the disease TNM, blood tests, imaging (mammography, ultrasound, CT-Scan, PET), tolerability to treatment, usually accompanied by corticosteroids (Dexamethasone) and drugs to control symptoms (Ondansetron, etc.) to reduce adverse effects. **Cytotoxicity: Inhibition of DNA and RNA synthesis. Inhibition of topoisomerase II enzyme, generation of free oxygen radicals, Induction of histone eviction from chromatin etc.

**Table 2:** Nanomedicine for triple negative breast cancer theranostics.

Nanoparticle	Unique Properties	Application	Status	Evidence
Quantum dots (QDs)	Semiconductor nanocrystals they have superior light absorbance and high fluorescent intensity [181].	QD-based nanotechnology possesses wider applications in cancer molecule imaging and quantitative detection.	Experimental/clinical ongoing	Many studies signs QD technology could substitute immunohistochemistry (IHC) [178], because of its better fluorescent signaling, and performing even more accurate quantitative analyses for evaluating prognosis for triple-negative breast cancer cells (TNBCs) [181].
Fluorescent nano-diamonds (FNDs)	Tunable-enhanced optoelectronics features allows fluorescent nano-diamonds (FNDs) issuing image signals [156] at low-cost production.	Current nuclear medicine uses radioisotopes such as strontium-89, iodine-131, samarium-183 and technetium-99. FNDs proposes the use of non-radioactive materials for imagining applications enhancing sensitivity and specificity.	Experimental/clinicalongoing	Fluorescent nano-diamonds (FNDs) are biocompatible nanomaterials often used in MDA-MB-231 theranostics [183].
Nano-matryoshkas	Nano-matryoshka, referred to as a multi-layer nanoparticle, hollow nanoparticles can deliver multiple drug payloads.	Nano-matryoshka, singular design has been developed as thermal therapeutic, imaging and drug-delivery nanoparticles.	Experimental/clinicalongoing	Designed by multilayers that can be designed with different materials, Nano-matryoshka can exert several drug medication payloads and inducing hyperthermia as suggest an MDA-MB-231 murine xenograft study [184].
Silver nanoparticles (AgNPs)	The mechanism of action is physical. However, it has not been specifically established. Ag affects cellular microenvironment will lead to the release of reactive oxygen species.	Therapeutics by using cytotoxicity.	Experimental/clinicalongoing	Silver NPs (AgNP) are another example of Nps that can act against tumor cells in TNBC that can induce DNA damage as in vivo studies suggest. Silver nanoparticles help in reduction of TNBC growth and augments radiation therapy. [169].
Iron oxide nanoparticles (IONP)	Tunable-enhanced optoelectronics and magnetic features.	The ability of iron oxide NPs to produce strong contrast images in MRI in T1(longitudinal relaxation—spin-lattice) and T2 (transversal relaxation—spin-spin) has given them a place in the theranostic of Cancer [9,90].	Experimental/clinicalongoing	This novel imaging system by using IONP has been used in several xenograft models [193,194], for MRI diagnostic on TNBC [195].
SPIONs (superparamagnetic iron oxide nanoparticles)	SPIONs have higher magnetic properties than paramagnetic materials due to their ability to spin alignment to an external magnetic field	SPIONs can generate heat inside the tumors producing apoptosis by using hyperthermia as well as real time images into the tumors [195]	Experimental/clinicalongoing	SPIONs are often use in human triple-negative breast cancer cells (TNBC) MDA-MB-231 therapeutics [209].
Core-shell nanoparticles	SPIONs core-shell are formed by layers: a magnetic iron oxide core and a therapeutic biocompatible coating [192] which can reduce toxic side effects [194].	Enhanced hyperthermia properties, by stimulation through lasers [196], ultrasound [197], radio frequencies [193] or alternating magnetic field [199] to generate apoptosis.	Experimental/clinicalongoing	Core shell design has been used for enhancing photodynamic, chemotherapy and gene therapy in TNBC [210]. Also Hayashi et al. [195] have shown in advantages of using SPION intravenously for cancer theranostics.
Gold nano-stars	Enhanced optoelectronics specifically T1-signal for RMI.	Theranostics Gene Therapy Photodynamics Drug delivery Hyperthermia Drug Delivery	Experimental/clinicalongoing	RMI T1- signal magnetic resonance imaging and photothermal therapy for TNBC [211].
Nanocages	Capacity to transport and deliver nucleic acids, peptides and drugs as well as PDT properties.	Theranostics Gene Therapy Immunotherapy Photodynamics Hyperthermia Imaging	Experimental/clinicalongoing	Immunogenic photodynamic therapy with gold nanocages on TNBC [212].
Nanorods	Enhanced magnetic-optoelectronics properties according to shape and size. Capacity to transport and deliver nucleic acids, peptides and drugs.	Theranostics Gene Therapy Immunotherapy Photodynamics Hyperthermia Imaging Drug Delivery	Experimental/clinicalongoing	Gold nanorods were developed for delivering cisplatin and producing photothermal therapy on TNBC [213].
Nanocomposites	Enhanced magnetic-optoelectronics including plasmon surface resonance properties. Nucleic acids, peptides and drug releasing with enhanced specificity.	Theranostics Gene Therapy Immunotherapy Photodynamics Hyperthermia Imaging Drug Delivery	Experimental/clinicalongoing	Researchers are experimented on using immunotherapy nanocomposites vehicle on TNBC [214].
